# Influence of Discharge Current on Phase Transition Properties of High Quality Polycrystalline VO_2_ Thin Film Fabricated by HiPIMS

**DOI:** 10.3390/ma10060633

**Published:** 2017-06-09

**Authors:** Tiegui Lin, Jian Wang, Gang Liu, Langping Wang, Xiaofeng Wang, Yufen Zhang

**Affiliations:** 1 State Key Laboratory of Advanced Welding and Joining, Harbin Institute of Technology, Harbin 150001, China; 13b309015@hit.edu.cn (T.L.); apxfwang@hit.edu.cn (X.W.); 13b309005@hit.edu.cn (Y.Z.); 2 School of Electrical and Electronic Engineering, North China Electric Power University, Beijing 102206, China; 13261201625@163.com; 3 Research and Development Center, Shanghai Institute of Spacecraft Engineering, Shanghai 200240, China; elest@126.com

**Keywords:** VO_2_ thin film, high power impulse magnetron sputtering, discharge current, phase transition, crystalline orientations, grain size

## Abstract

To fabricate high-quality polycrystalline VO_2_ thin film with a metal–insulator transition (MIT) temperature less than 50 °C, high-power impulse magnetron sputtering with different discharge currents was employed in this study. The as-deposited VO_2_ films were characterized by a four-point probe resistivity measurement system, visible-near infrared (IR) transmittance spectra, X-ray diffraction (XRD), X-ray photoelectron spectroscopy (XPS), and scanning electron microscopy. The resistivity results revealed that all the as-deposited films had a high resistance change in the phase transition process, and the MIT temperature decreased with the increased discharge current, where little deterioration in the phase transition properties, such as the resistance and transmittance changes, could be found. Additionally, XRD patterns at various temperatures exhibited that some reverse deformations that existed in the MIT process of the VO_2_ film, with a large amount of preferred crystalline orientations. The decrease of the MIT temperature with little deterioration on phase transition properties could be attributed to the reduction of the preferred grain orientations.

## 1. Introduction

Vanadium dioxide has thermochromic properties associated with a reversible metal–insulator transition (MIT) at 68 °C, where its structure is changed from an insulating monoclinic phase to a metallic tetragonal one [1]. The high-temperature phase VO_2_(R) is metallic with a rutile crystalline structure (R, P42/mnm). The low-temperature phase VO_2_(M) is characterized by the semiconducting electronic behavior (with a band gap energy Eg ≈ 0.6 eV) and monoclinic structure (M1, P21/c) [2]. The MIT of VO_2_ results in a remarkable change in physical characteristics, including the resistance and transmittance [2,3], making it a potential candidate for many applications, such as optical switching, thermochromic smart windows, laser protection, and energy harvesting systems [4,5,6].

VO_2_ thin films have been fabricated by a variety of deposition techniques, such as reactive sputtering [7,8,9,10,11,12,13], pulsed laser deposition (PLD) [14,15], molecular beam epitaxy (MBE) [16], sol-gel processing [17], thermal oxidation [18,19], and reactive electron beam evaporation [20]. Previous studies have discussed the transition properties of VO_2_ thin films extensively, such as the phase transition temperature (*T*_MIT_) [10,21,22,23,24], hysteresis width [25,26,27], resistance change, and sharpness of the transition [1,26,27,28]. Kim et al. [1] showed that the resistance change of the epitaxial VO_2_ thin film on single-crystal c-plane sapphire substrates reached about four orders of magnitude, and the hysteresis width, as well as sharpness of the transition, were very small. However, a significant deterioration on the resistance change took place with the reduction of *T*_MIT_, and it could be reduced by 2000 times when the MIT temperature was decreased from 70.7 to 65 °C [1]. Fortunately, the MIT temperature of the epitaxial VO_2_ thin film could be reduced by using different substrates, and high-quality epitaxial VO_2_ thin film with a MIT temperature of 30 °C was prepared on TiO_2_ (001) substrate [29]. For polycrystalline VO_2_ thin films, according to their phase transition properties [26,27,28,30,31,32], it can be concluded that a slight reduction of the MIT temperature will lead to a large deterioration of the electrical and optical properties. Until now, fabrication of high-quality polycrystalline VO_2_ thin film with a *T*_MIT_ less than 50 °C and phase transition properties near those of the epitaxial VO_2_ thin film is still a significant challenge.

In a previous study, we reported that polycrystalline VO_2_ thin film with a resistance change of 4500 times, a hysteresis width of 6 °C, a sharpness of the transition 5.8 °C, and an MIT temperature of 54.5 °C was prepared successfully on quartz substrates by high-power impulse magnetron sputtering (HiPIMS). However, the electrical and optical properties of this polycrystalline VO_2_ thin film deteriorated significantly with the decrease of the MIT temperature, and the resistance change was only 120 times when the phase transition temperature was reduced to 48.3 °C [33,34]. Meanwhile, we deduced that the better way to reduce *T*_MIT_ with only a slight deterioration on phase transition properties was to fabricate VO_2_ thin film with a high ordered crystalline orientation close to that of the epitaxial VO_2_ thin film. Since HiPIMS can generate dense plasma and intense ion impingement during the deposition process [35,36,37,38], in which the plasma density can be changed by different discharge currents, the crystalline structure of the deposited VO_2_ thin film may be improved by a proper discharge current [39]. 

In this study, polycrystalline VO_2_ thin films were fabricated on quartz substrates using HiPIMS with various discharge currents, and influences of the discharge current on the phase transition properties of the films were discussed. 

## 2. Experimental Detail

**Figure 1:**
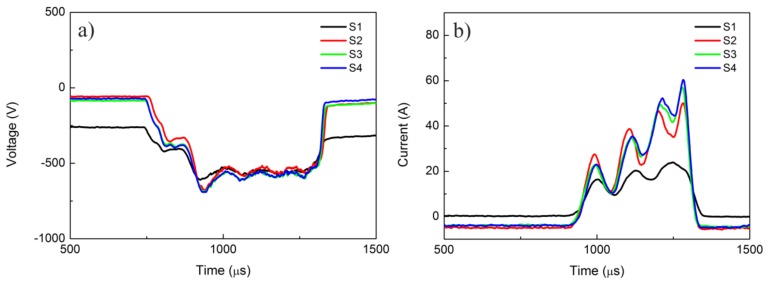
Cathode (**a**) voltage and (**b**) current waveforms for different samples.

VO_2_ thin films were fabricated on quartz substrates (30 × 30 × 1 mm^3^) using HiPIMS in Ar + O_2_ gases. A vanadium target with a purity of 99.95% and diameter of 100 mm was utilized for the fabrication process, and the distance between the target and the substrate was about 100 mm. Prior to deposition, the vacuum chamber was evacuated to a base pressure of below 1 × 10^−3^ Pa by using mechanical and molecular pumps, and the quartz substrate was heated to 420 °C. During the HiPIMS process, the mixture of Ar + O_2_ was introduced into the vacuum chamber of the HiPIMS apparatus, and the corresponding mass flow rates were 100 and 6.5 sccm, respectively. The operating pressure in the vacuum chamber during the deposition process was kept at about 0.85 Pa. In order to increase the deposition rate, we used a middle frequency modulated HiPIMS power supply for this process, and the magnitude of the peak discharge current was varied from 25 to 62 A. Figure 1 shows the discharge voltage and current waveforms for different samples. According to these waveforms, the peak currents for samples S1, S2, S3, and S4 were 25, 51, 58, and 62 A, respectively. Main processing parameters for different samples are listed in Table 1. The whole deposition time was 30 min, and the thicknesses of samples S1, S2, S3, and S4 were about 164, 119, 122, and 115 nm, respectively.

The structures of VO_2_ thin films were characterized using X-ray diffraction (XRD; Empyrean, Panalytical, Almelo, The Netherlands) at a grazing angle of 1° with a Cu-Kα radiation source operated at 40 kV and 40 mA. The compositions of the as-deposited thin film were detected by X-ray photoelectron spectroscopy (XPS, ESCALAB 250Xi, Thermo Fisher Scientific, Waltham, MA, USA). The surface morphology of the as-deposited films was examined by a field emission scanning electron microscope (SEM; Quanta 200, FEI Co., Hillsboro, OR, USA) with an acceleration voltage of 5 kV. Surface roughness of the as-synthesized samples was measured by an atomic force microscope (AFM, Dimension Icon, Bruker Co., Karlsruhe, Germany) with a scanning range of 5 × 5 μm^2^. Optical transmittance spectra were obtained by using a Lambda 950 spectrophotometer (PerkinElmer Co., Waltham, MA, USA) at 24 and 75 °C. A four-point probe resistivity measurement system was used to measure the electrical resistance of the film and its variation with the temperature.

## 3. Results and Discussion

**Figure 2:**
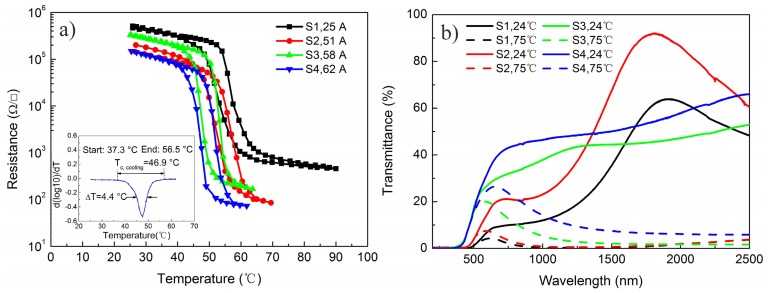
(**a**) Temperature dependencies of the electrical resistance for the as-deposited VO_2_ thin films; and (**b**) the transmittance spectra of the as-deposited VO_2_ films at various temperatures.

The temperature dependences of the resistance for all the as-deposited VO_2_ thin films are shown in Figure 2a. A derivative curve (*d[log(R)]/dT*) during the cooling process of sample S4 was inserted into this Figure. Therein, the maximum resistance before the phase transition (*R*_0_), minimum resistance after the phase transition (*R*_1_), start temperature (*T*_start_), end temperature (*T*_end_), transition temperatures during cooling and heating (*T*_c__, cooling_ and *T*_c__, heating_) could be obtained from the derivative curves. The relative resistance change (*∆R*), transition temperature (*T*_MIT_), sharpness of the transition (*∆T*) and hysteresis width (*∆H*), were defined according to our previous study [33]. Phase transition characteristics of the as-deposited VO_2_ thin films are listed in Table 2. Note that all as-deposited thin films showed large resistance changes and narrow hysteresis widths, and the MIT temperature of the as-deposited VO_2_ thin film was varied from 55.7 to 49.2 °C when the peak discharge current was increased from 25 to 62 A. Particularly for sample S4, although the MIT temperature of this film was reduced to 49.2 °C, the resistance change, hysteresis width, and sharpness of the transition were 2100, 4.6 °C and 4.4 °C, respectively, which were close to those of the epitaxial VO_2_ thin films [1,21]. Consequently, reduction of the MIT temperature of VO_2_ thin film with less deterioration on phase transition properties was obtained by this method, and the MIT temperature was reduced to less than 50 °C when the discharge current was greater than 58 A.

The transmittance spectra of the as-deposited VO_2_ films at various temperatures are exhibited in Figure 2b. It is clear that the transmittance of the as-deposited VO_2_ thin film in near-infrared region decreased greatly when the temperature was increased from 24 to 75 °C, which proves that all of the as-deposited VO_2_ films possessed representative metal–insulator transitions. Moreover, the transmittance curves of samples S1 and S2 were similar, and their transmittance changes at 2500 nm were 44.6% and 56.7%, respectively. For samples S3 and S4, the transmittance curves were different from those of samples S1 and S2, and the transmittance changes at 2500 nm were 51.3% and 60.1%, respectively. Especially for sample S4, the transmittance curve was similar to that of the epitaxial VO_2_ film [1], which implies that the structure of sample S4 should be a perfect one.

**Figure 3:**
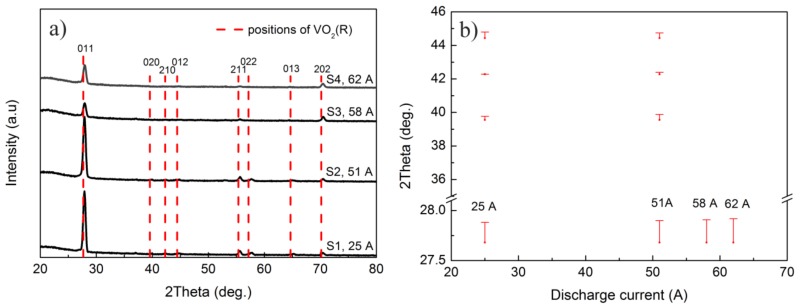

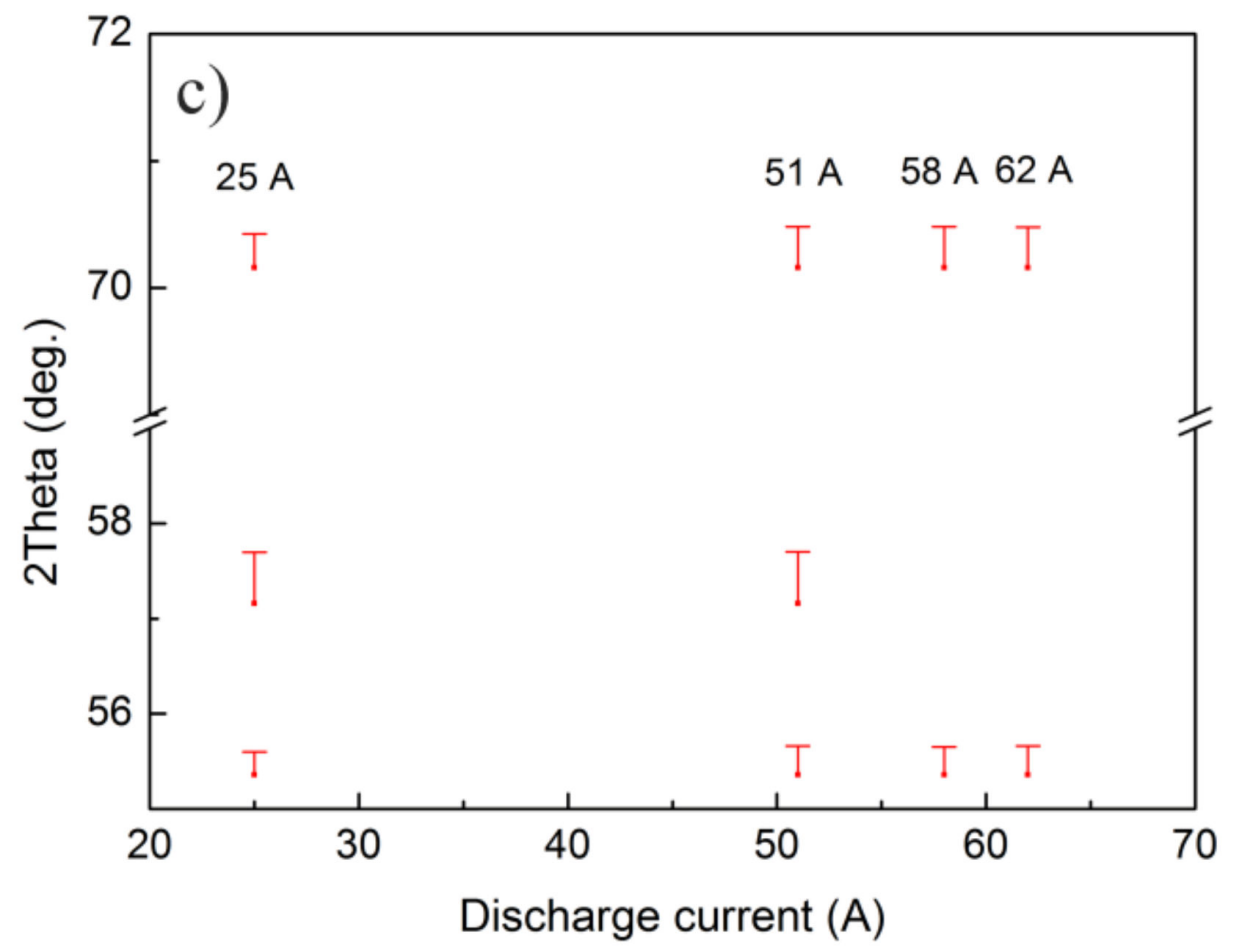
(**a**) X-ray diffraction (XRD) patterns of the as-deposited VO_2_ thin films grown on quartz substrates; and (**b**,**c**) the magnified 2θ differences between the experimental diffraction angles of the as-deposited films and those of the standard VO_2_(R).

XRD patterns of the as-deposited VO_2_ thin films are shown in Figure 3a. The obvious (011), (211), and (202) peaks in the patterns confirmed that all the as-deposited VO_2_ thin films were polycrystalline VO_2_(M) (JCPDS 44-0252). In addition, an intense diffraction peak of the VO_2_ (011) plane was prominent for all films. More than five diffraction peaks, such as (011), (020), (210), (211), and (022) etc., could be found in the XRD patterns of samples S1 and S2, while only (011), (211), and (202) peaks were conspicuous in those of samples S3 and S4. Therefore, it can be deduced that a large discharge current benefits the growth of high-ordered polycrystalline VO_2_ thin film.

According to previous studies, the MIT temperature of VO_2_ thin film can be affected by a lattice distortion, and the transition temperature decreases with the difference between the interplanar spacing of the as-deposited thin film and standard rutile VO_2_ [29,33,40]. To compare the lattice distortions before and after the phase transition process, the respective diffraction angles of standard VO_2_(R) (JCPDS 44-0253) were marked with red dashed lines. Since the magnitudes of the 2θ deviations are not clear in Figure 3a, magnified 2θ differences between the experimental diffraction angle of the as-deposited film and that of the standard VO_2_(R) are shown in Figure 3b,c. It can be found that the 2θ deviations were basically the same when the discharge current was changed, which means that the lattice distortions in all of the as-deposited films were similar. Therefore, the influence of the lattice distortion on the phase transition temperature of these films is neglected in this work. 

**Figure 4:**
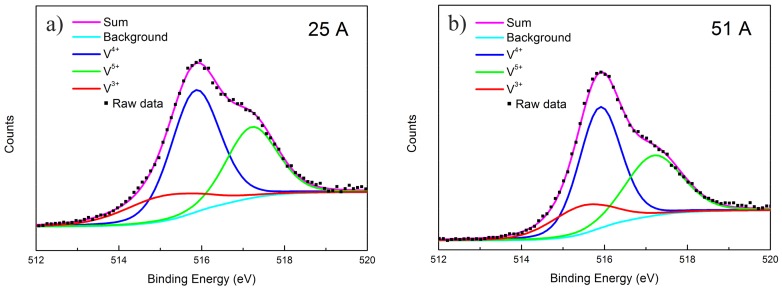

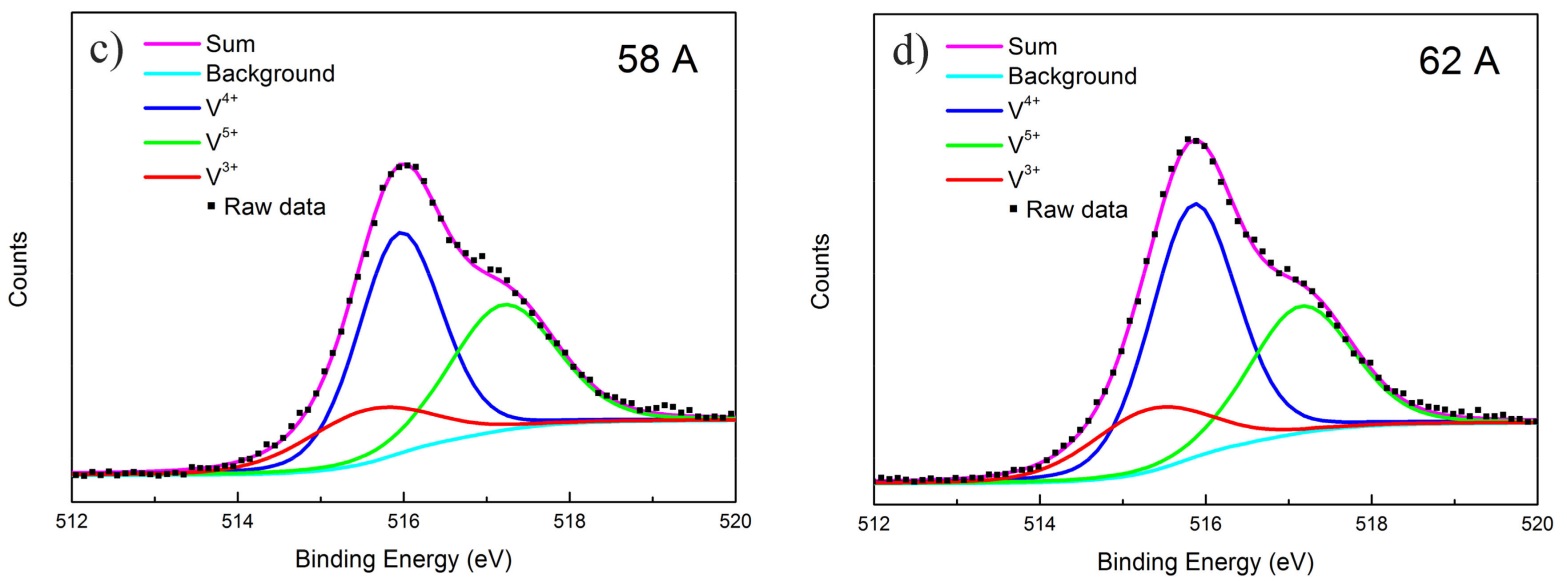
X-ray photoelectron spectroscopy (XPS) V(2P) spectra for different as-deposited VO_2_ thin films. (**a**) S1; (**b**) S2; (**c**) S3; and (**d**) S4.

The XPS V(2p) spectra recorded on the as-deposited VO_2_ film are exhibited in Figure 4a–d. All of the peaks were resolved into three main components at 516.9–517.2, 515.7–516.2, and 515.2–515.7 eV, corresponding to V^5+^, V^4+^ and V^3+^, respectively [41]. In addition, the relative concentration of V^3+^, V^4+^ and V^5+^ in the as-deposited VO_2_ thin films were calculated and are listed in Table 3. It is clear that VO_2_ was the main content for all the films, and the contents of V^5+^, V^4+^ and V^3+^ were similar. According to previous studies [41,42,43], V^5+^ could be attributed to the surface oxidization during storage in air, and the occurrence of V^3+^ was to keep the charge neutrality in the VO_2_ thin film.

**Figure 5:**
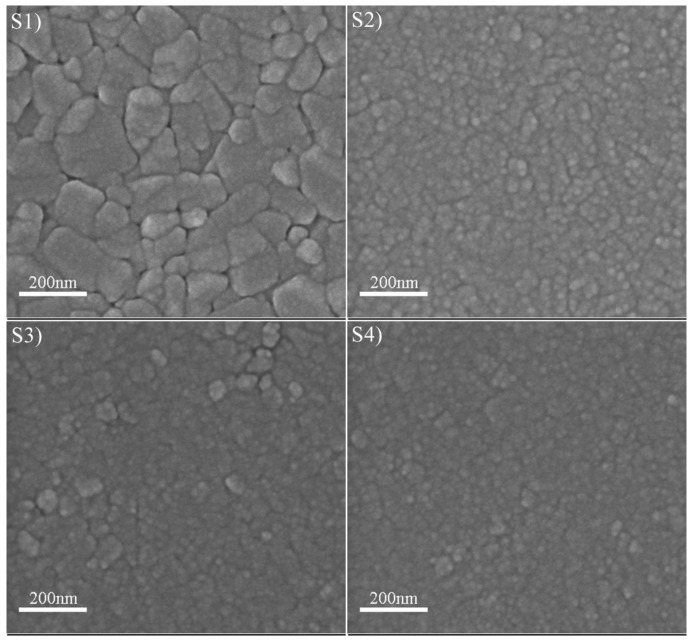
Scanning electron microscope (SEM) images of as-deposited VO_2_ thin films.

**Figure 6:**
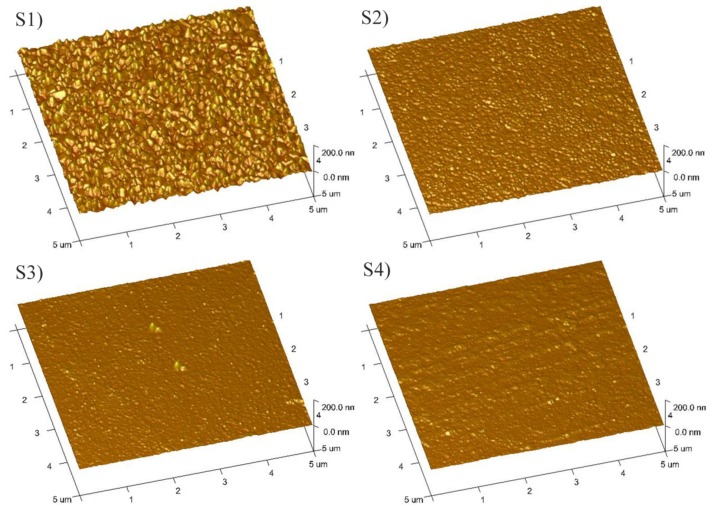
Atomic force microscope (AFM) images of as-deposited VO_2_ thin films.

Figure 5 displays the SEM images of the as-deposited VO_2_ thin films. It is clear that all the films possessed a nano-crystalline structure. In addition, the grain size varied with the discharge current. It can be found that sample S1 had the largest grain size, and other samples possessed similar small grains. As we know, the greater the peak current, the higher the ion density, and a high density ion impingement will retard the growth of the grains. Therefore, a large discharge current benefits the reduction of the crystalline size. However, when the discharge current was greater than 51 A (samples S2, S3, and S4), the crystalline size did not decrease any longer. The AFM images of the as-deposited VO_2_ thin films are shown in Figure 6. The roughness of samples S1, S2, S3, and S4 was 7.39, 2.14, 1.45, and 1.76 nm, respectively, which coincided with the SEM results.

Other studies have proved that the grain size affects the phase transition temperature of the polycrystalline VO_2_ thin film, and the film with a small gain size possessed a low MIT temperature [23,26,44]. As a result, the high MIT temperature of sample S1 could be attributed to its large grain size. However, since the grain sizes of samples S2, S3, or S4 were similar, the influence of the grain size on the phase transition temperature of these samples could be neglected. Similarly, the impact of the thickness could be ignored as well because these films possessed similar thicknesses.

**Figure 7:**
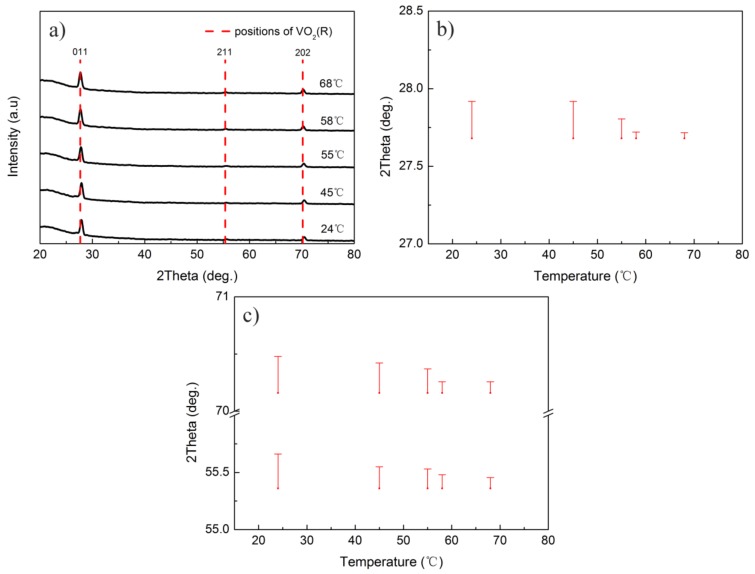
(**a**) XRD patterns of sample S4 during the heating process, and (**b**,**c**) magnified 2θ differences between the experimental diffraction angles and those of the standard VO_2_(R) at different temperatures.

To explain the reason that the MIT temperature of sample S2 was higher than those of samples S3 and S4, the structural changes in phase transition processes of samples S4 and S2 were compared. Figure 7a shows the XRD patterns of sample S4 during the heating process. The red dashed lines marked the diffraction peaks of standard VO_2_(R). Figure 7b,c give the magnifications of the deviations in 2θ from the peaks of standard VO_2_(R). It is clear that the interplanar spacing of (011), (211) and (202) planes of sample S4 was shifted step by step towards those of the standard VO_2_(R) in the heating process, implying that the crystal structure of polycrystalline VO_2_(M) thin film gradually became more similar to that of VO_2_(R). However, the change in the diffraction angle of the (011) plane was very small when the temperature was increased from 24 to 45 °C, which hints that the crystalline structure of the as-deposited film kept its original configuration in this temperature region. When the temperature was elevated from 45 to 58 °C, an obvious change in 2θ took place. When the temperature was increased from 58 to 68 °C, the reduction of the diffraction angle was very slight. Meanwhile, the diffraction peaks of the as-deposited VO_2_ thin film at 68 °C basically coincided with those of standard VO_2_(R), which reveals that the phase transition was completed. In addition, from the temperature dependences of the electrical resistance for sample S4, we can also find that the resistance changed rapidly from 45 to 58 °C, which should be derived from the obvious structural change in this region.

**Figure 8:**
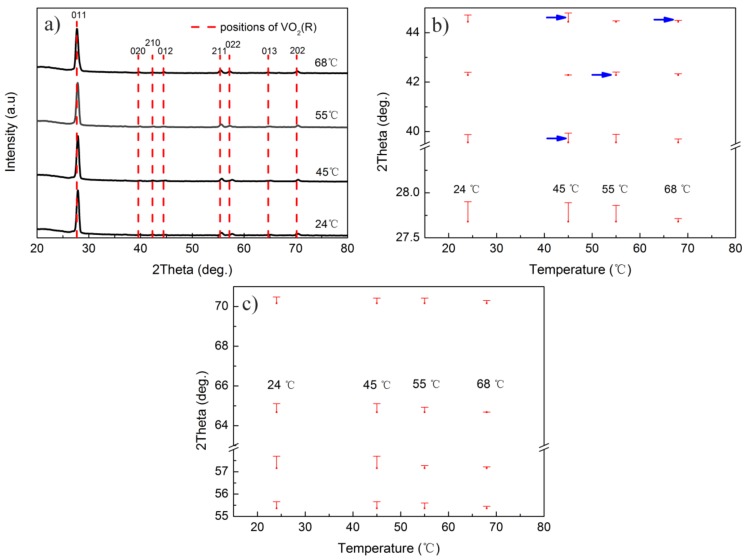
(**a**) XRD patterns of sample S2 during the heating processes; and (**b**,**c**) magnified 2θ differences between the experimental diffraction angles and those of the standard VO_2_(R) at different temperatures. Therein, the diffraction angles indicated by the blue arrows increased instead of decreasing during heating process.

The XRD patterns of sample S2 at different temperatures during the heating process are shown in Figure 8a. Magnifications of the deviations in 2θ are shown in Figure 8b,c. During the phase transition process of sample 2, the interplanar spacing changes of (011), (211), (013), and (202) planes were similar to those of sample S4. However, as indicated by the arrows in Figure 8b, the interplanar spacing of the (020), (210), and (012) planes decreased rather than increased during the heating process, which means that some grains in sample S2 possessed reverse deformations during the heating process. Additionally, this phenomenon was also shown in Peter’s [13] study, in which the interplanar spacing of the (011) plane in VO_2_ film with a thickness of 30 nm was decreased when the temperature was increased from 20 to 60 °C.

Based on above analysis, we can find that some reverse deformations existed in the phase transition process of the VO_2_ thin film with a large amount of crystal orientations, and its probability decreased with the quantity of the crystal orientations. Obviously, an opposite deformation along the preferred crystalline orientation will retard the phase transition process, while a similar deformation will benefit such a process. Comparing the XRD patterns of samples S2 and S4 at different temperatures, we can deduce that similar deformations of different grains during the phase transition process benefit the reduction of the *T*_MIT_. Consequently, fabrication of the VO_2_ thin film with a small grain size and fewer crystalline orientations is a better method to reduce *T*_MIT_ with little deterioration of the phase transition properties.

## 4. Conclusions

Polycrystalline VO_2_ thin films were prepared on the quartz substrates successfully by HiPIMS with the peak discharge current varied from 25 to 62 A. The microstructure and phase transition properties of the as-deposited films were influenced by the current. When it was 62 A, high-quality VO_2_ thin film with a resistance change of 2100 times, an MIT temperature of 49.2 °C, a transmittance change of 60.1%, a hysteresis width of 4.6 °C, and a transition sharpness of 4.4 °C, was obtained. The excellent phase transition properties were derived from the small grain size and high ordered preferred orientations in the film, in which reverse deformations did not exist during the phase transition process.

## Figures and Tables

**Table 1:** Main processing parameters for different samples.

Sample No.	Discharge Voltage (V)	Peak Current (A)	Pulse Frequency (Hz)	Pulse Width (μs)	Average Power (W)
S1	640	25	100	400	403
S2	670	51	50	400	385
S3	680	58	50	400	401
S4	683	62	50	400	403

**Table 2:** Phase transition characteristics of the as-deposited VO_2_ thin films.

Sample No.	*T*_c, heating_ (°C)	*T*_c__, cooling_ (°C)	*T*_MIT_ (°C)	*∆H* (°C)	*∆T* (°C)	*R*_0_ (Ω/□)	*R*_1_ (Ω/□)	*∆R*
S1	58	53.4	55.7	4.6	8.5	5.2 × 10^5^	4.7 × 10^2^	1.1 × 10^3^
S2	55.6	51	53.3	4.6	7.4	2 × 10^5^	90	2.2 × 10^3^
S3	52.8	47.2	50	5.6	4.6	3.1 × 10^5^	1.9 × 10^2^	1.6 × 10^3^
S4	51.5	46.9	49.2	4.6	4.4	1.5 × 10^5^	70	2.1 × 10^3^

**Table 3:** Content of V with different valence states.

Sample No.	V^3+^	V^4+^	V^5+^
S1	17.60%	51.38%	31.02%
S2	17.14%	49.69%	33.17%
S3	17.07%	48%	34.93%
S4	17.30%	51.80%	30.90%
